# Complementary and Alternative Medicine Use in a Pregnant Population, Northwest Ethiopia

**DOI:** 10.1155/2021/8829313

**Published:** 2021-08-06

**Authors:** Yohannes Kelifa Emiru, Betelhem Anteneh Adamu, Melak Erara, Tigist Chanie, Abyot Endale Gurmu

**Affiliations:** ^1^Department of Pharmacognosy, School of Pharmacy, College of Medicine and Health Sciences, University of Gondar, Gondar, Ethiopia; ^2^Department of Clinical Pharmacy, School of Pharmacy, College of Medicine and Health Sciences, University of Gondar, Gondar, Ethiopia; ^3^University of Gondar Compressive Specialized Hospital, University of Gondar, Gondar, Ethiopia

## Abstract

**Background:**

Complementary and alternative medicine (CAM) appears to be the source of healthcare particularly in the majority of pregnant communities of Africa due to its intrinsic qualities as well as its accessibility and affordability. Despite acknowledged benefits of CAM use in pregnancy, majority of users are unaware of its safety and effects on fetal development. The present study was aimed at examining CAM use among pregnant women in Northwest Ethiopia to provide an opportunity for future investigations on the effectiveness of CAM modalities in the management of pregnancy-related complications across the country.

**Methods:**

This was an institution-based cross-sectional study which was conducted at Poly Clinic Health Center in the Northwest part of Ethiopia between March and May 2018. Two hundred and eighty two Ethiopian women were conveniently recruited to take part in the study. Structured questioners were used for the survey. Descriptive statistics of sociodemographic and CAM use characteristics were expressed in frequencies and proportions. Chi-square test was performed to determine the difference between CAM user and nonuser. Besides, binary logistic regression analyses were performed to examine predictors of CAM use in the study population. The result was considered statistically significant if *P* value ≤ 0.05.

**Results:**

The prevalence rate of CAM use in pregnant women was 89.36% with the commonest practice of spiritual healing (65.2%) and herbal supplement (51.8%) CAM therapies. Use of CAM positively associated with marital status and previous number of children. The odds of using CAM among single/not married women were 3.22 times higher (COR: 3.22, CI: 1.07-9.64) as compared to married women. Pregnant women with no children were 3.30 times more likely (COR: 3.30, CI: 0.92-11.84) to use CAM than those women having ≥3 children. Lower odds of using CAM significantly associated with educational level (COR: 0.20, CI: 0.046-0.93) and antenatal care (COR: 0.45, CI: 0.18-1.13) in a binary logistic regression model.

**Conclusions:**

A considerable number of pregnant women were utilizing CAM including herbal products as part of their maternity care. This finding provides a reference on the use of CAM for policy-makers, health professionals, and parents. Further studies are needed to investigate the effectiveness and safety of specific CAM modalities with particular focus on herbal medicinal products.

## 1. Introduction

In resource-constrained health settings, complementary and alternative medicines (CAM) are commonly used because they are often more widely available and more affordable than conventional therapies. The term CAM refers to a wide set of healthcare practices that are not integrated into the current healthcare system and are not part of that country's tradition [1]. It comprises some form of caring approach including acupuncture, especial diets, massage therapy, medicinal plants, biologic feedback, and relaxation techniques [1, 2]. Women have become more informed about their health and use print media, television, radio, and internet to get information on which to base their health decisions. As a result, pregnant women are increasingly recognized CAM and they are leading the utilization trends of CAM for their healthcare and well-being [2, 3]. In the United States of America [4, 5] and Australia [6], for instance, they reported a practice rate of 87% and 91% CAM use during pregnancy, respectively.

Studies revealed the use of CAM throughout the pregnancy period for the treatment of nausea and vomiting, as well as for illnesses due to pregnancy such as fatigue, respiratory, and skin issues and nutritional benefits [7, 8]. Despite known benefits of CAM use in pregnancy, majority of users are unaware of its safety and effects on fetal development and possibility of CAM-drug interaction [1, 7, 9]. Together with this, a substantial proportion of women often fail to disclose their gestational use of CAM to their maternity providers [6, 7]. In Ethiopia, CAM is culturally acceptable and widely utilized, predominantly for the belief that CAM is natural, thus safer than conventional medicine. A previous a study done in Northwest Ethiopia has shown that higher than 48% pregnant women use herbal medicine like ginger (*Zingiber officinale*) and demakese (*Ocimum lamiifolium*) [8]. However, there is paucity of evidence on the prevalence and predictors of other types of CAM modalities such as spiritual healing, biological-based therapies, and manipulative and body-based therapy practices among pregnant women in the nation. Hence, this study was aimed at investigating the prevalence, types, and sociodemographic factors associated with CAM usage among pregnant women in Northwest Ethiopia. The findings of this study will provide supporting evidence that could guide decision making at the system, institutional, and individual level concerning CAM use in pregnancy and provide an opportunity for future investigations on the effectiveness of these modalities in the management of pregnancy-related complications across the country.

## 2. Methods and Materials

### 2.1. Study Design and Setting

This was an institution-based cross-sectional study which was carried out at Poly Clinic Health Center (PCHC) from March to May 2018. The source population was pregnant women who attended antenatal care clinics. The study area is found in Gondar Town, North Gondar, which is located 738 km far away from Addis Ababa, capital of Ethiopia. The town is found at 2700 meters above sea level and has a total population of 333,103. PCHC is one of the governmental health centers serving for 101,317 populations.

### 2.2. Sample Size and Sampling Procedure

A convenience sampling technique was used in this study. Eligible participants had to meet a set of inclusion criteria recruited in the study. These criteria are Ethiopian nationality only (as this study was intended to explore CAM use particularly in Ethiopian population); age ≥ 18 years, willing to participate, and who had provided verbal consent to participate in the study; and completely filled the questionnaire form and answered all questions. Pregnant women who had severe physical and mental illness were excluded.

### 2.3. Data Collection Tool

The survey questionnaire used in the study was adapted and developed following a detailed review of previous relevant works [1, 8, 10] with some modifications. The questionnaire was constructed in their native language (Amharic) and translated back into English to check its consistency. The content of the questionnaire was piloted among 15 pregnant women visiting Maraki Health Center (one of the public health centers found in Gondar Town) to gather information on its understandability, time consumed by each question, consistency among related variables, and acceptability. Following the results of the pre-test, necessary correctionsand amendments were made on the questionnairebefore the actual data collection was initiated The respondents were interviewed using a structured questionnaire which consists of 4 parts: part I: eleven questions related to sociodemographic characteristic of the respondents; part II: six practice-related questions for CAM users; part III: eleven questions asking the perceived reasons for CAM use; and part IV: four queries demanding types of CAM therapies frequently practiced in the course of pregnancy.

### 2.4. Ethical Approval

Ethical clearance was obtained from the ethics review committee of School of Pharmacy, College of Medicine and Health Science, University of Gondar. Additionally, the permission to undertake the study was received from the PCHC director. The study subjects were informed about the objective and importance of the study, and oral consent was obtained from each participant. All respondents were assured of the confidentiality regarding the responses obtained from them.

### 2.5. Statistical Analysis

Statistical analysis was performed using Statistical Package for the Social Sciences (SPSS) version 22.0 for Windows. Descriptive statistics of sociodemographic and CAM use characteristics were expressed in frequencies and proportions. The main outcome variable in the analysis was CAM use. In order to determine the differences in characteristics between pregnant women who have used CAM and those who did not, chi-square test was performed. Moreover, the univariate and multivariate logistic regression analyses were performed to examine predictors of CAM use in the study population. For a variable to be included in the multiple regression model, it had to be significantly associated with the main outcome in the univariate analysis. Odds ratios and their respective 95% confidence intervals were calculated. Statistical significance was set at a *P* value ≤ 0.05.

## 3. Results

### 3.1. Characteristics of CAM and Non-CAM User Pregnant Women

Among the 282 pregnant subjects who had attended the antenatal care, 252 (89.36%) pregnant women reported to be using some form of CAM modalities (Table 1). The vast majority (90.8%) were married, and only 11% had attended college/university, whereas 27% had secondary education and 27.7% were unable to write and read. Most of the participants were unemployed (57.4%), and only 33% were earning more than Ethiopian birr (ETB) 1000 per month (≈United States Dollar (USD) 32), while 30.5% and 29.4% had a monthly income of ETB < 500 and 500-99, respectively. In respect of pregnancy status, 44% of women were found in 2^nd^ trimester and majority (74.1%) had visited midwifery for the antenatal care.

In a univariate logistic regression model, those who were single had higher odds of using CAM (COR: 3.22, CI: 1.07-9.64) compared to married or divorced or widowed women. Similarly, no children in previous time showed higher odds of CAM use (COR: 3.30, CI: 0.92-11.84). Pregnant women who were unable to write and read (COR: 0.20, CI: 0.046-0.93) and had visited midwifery for antenatal care (COR: 0.45, CI: 0.18-1.13) reported lower odds of using CAM. However, none of these variables was significantly associated with CAM use in multiple logistic regression analysis as shown in Table 1.

### 3.2. The Perceived Responses for Questions Linked to CAM Uses and Characterization of Types of CAM Used across Pregnancy Periods

The response in relation to CAM use among study subjects is presented in Table 2. Most of the CAM users were reported having slept well (65.6%) and less fatigue (62.4%) in response to CAM use-related quality of life (QOL) while a small proportion of participants responded having more energy (27.7%) and not sick all the time (26.6%) as CAM use-associated QOL. In the case of level of satisfaction in CAM use, 44.3% respondents had shown an average level of satisfaction for CAM therapies but about 56% of CAM users were not aware of any potential herbal-drug interaction. Majority of the pregnant women (55.7%) informed that they did not disclose CAM use to their HCPs. The reasons for not disclosing CAM use were reported to be 'HCPs do not ask' (20.2%), and 'HCPs may be unsupportive and discourage' (18.4%). Less reported reasons were insufficient information of CAM (6.7%) and CAM use does not need approval of HCPs (2.5%).

Regarding source of information for CAM use, nearly half of the participants relied 'family/friend/relative' (49.6%) while 14.2% answered 'health practitioner'. The utmost strongly agreed reasons for CAM use were accessibility and availability of CAM (28%), family/culture/religious beliefs (22%), more natural (17.4%), and the beneficial effect on quality of life and health of their fetus (15.2%) as described in Table 3.

**Figure 1:**
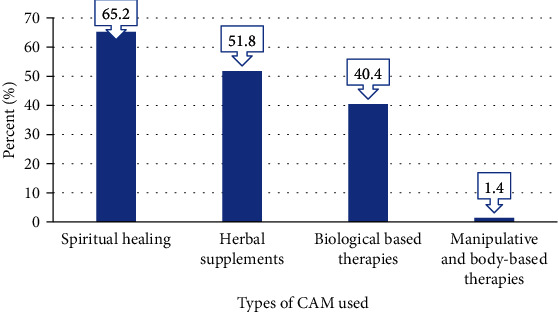
Frequency (%) and types of CAM therapies used during pregnancy.

**Figure 2:**
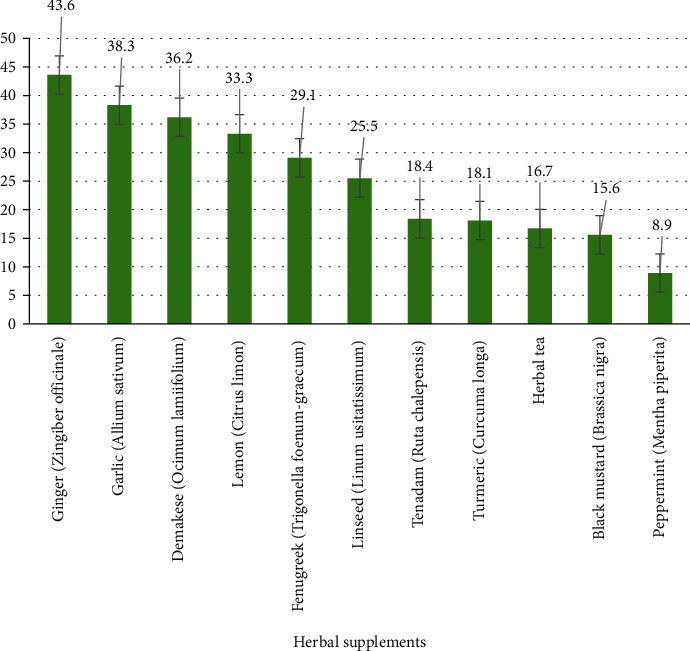
Frequency (%) and list of herbal supplements used during pregnancy.

Spiritual healing (65.2%) and herbal supplements (51.8%) were found to be the commonest types of CAM therapies used throughout pregnancy in the present survey as it is portrayed in Figure 1. The most frequently consumed herbal supplements were ginger (*Zingiber officinale*) (43.6%), garlic (*Allium sativum*) (38.3%), and demakese (*Ocimum lamiifolium*) (36.2%) (Figure 2).

## 4. Discussion

The present study was aimed at examining the prevalence and types of CAM therapies along with associated factors of CAM use during pregnancy. Despite a scarcity of evidence for efficacy and safety, CAM use is reportedly increasing across the globe [2, 3]. In this study, out of 252 women, 89.36% had reported CAM use at least once during pregnancy for strongly appreciated reasons like accessibility and availability of CAM treatments as well asculture/religion-related beliefs. Similarly, a study in Australia reported CAM use as high as 87% [11]. In contrast, one study done in the United Kingdom evidenced lower (26.7%) prevalence of CAM use in pregnancy. Moreover, three studies [12–14] from the USA reported lesser CAM use among pregnant women. The discrepancy in the prevalence of CAM use may be due to variation in sample size of participants surveyed, knowledge and experience of women, difference in advancement of health system across the countries, and scarcity in availability and access of medical services. In the current work, substantial increment of CAM use was observed in the first and second trimester in line with Mohamed et al.'s [15] study but a study undertaken in Qatar reported the relative decreases in the third trimester.

In this study, the commonest types of CAM modalities used during gestation were spiritual healing and herbal supplements followed by biological-based therapy. In accordance with previous studies conducted in the Northwest part of Ethiopia [8] and Alexandria [16], herbal supplements used by a higher proportion of pregnant women were *Zingiber officinale*, *Allium sativum*, and *Ocimum lamiifolium*. On the other hand, commonly used herbal supplements/preparations in Virginia, Australia, Norway, and Tuscany were reported to be peppermint, raspberry, fennel, and St. John's wort [17–20]. The inconsistency in the pattern of herbal supplement items between the studies might be due to availability, accessibility, affordability, distribution, and traditional claim/belief difference in the use of CAM for pregnancy-related medical conditions. Evidence on efficacy and safety of herbs indicated for pregnant populations is inadequate. Chamomile and peppermint for morning sickness for instance were shown risky in 6% of studies [21, 22]. *Zingiber officinale* and raspberry leaf were also evidenced as unsafe in 12% and 15% of the studies, respectively [21]. Fenugreek oil also needs to be consumed with caution during pregnancy due to its hypoglycemic effect and its stimulatory effect on oxytocin secretion resulting in uterine contraction [23]. The use of herbal products is not usually tested in clinical trials, and also, they are not included in the FDA pregnancy categories, giving a false impression of safety which could result in immense risk to the mother and fetus and elicit adverse effects including teratogenicity as well [24–26].

Concomitant use of herbs and conventional medications may result to undesired effects [27]. Remarkably, majority of pregnant women in the current study had possessed less awareness to herb-drug interaction while less proportion (33.7%) of women were reported to disclose CAM use to their HCPs. These results agree with the findings of other studies, in which 37.2% of pregnant women informed their HCPs [28]. Although these findings differ from a published study [29], they have reported that 65.8% of pregnant women disclosed uses of some form of CAM therapies to their physicians. Other studies also revealed that the reporting level of pregnant women about CAM uses to their HCPs ranges from 24% to 52% [30, 31]. Most importantly, results of the present study indicated 'HCPs' unsupportiveness and discouragement' and 'omission of HCPs to ask' were the major reasons for nondisclosure of CAM use. Other reasons that were speculated in a previous work [32] included fear and unwillingness to discuss with HCPs.

In this study, family/friends/relatives were found to be the most common sources of information to use CAM in agreement with John and Shantakumari et al.'s findings [33]. However, the findings of the current study do not support the previous researches which showed the health practitioner as a source of information [34–36]. The current study confirmed the association of CAM use with marital status and number of children previously. The odds of using CAM among single/not married pregnant women were 3.22 times higher as compared to married women. Pregnant women with no children were 3.30 times more likely to use CAM than those women having ≥3 children. Contrary to earlier findings [8, 37–40], this study indicated that lower odds of CAM use associated with educational level and antenatal care. Several limitations to this study need to be acknowledged while interpreting the results. First, the study did not identify a causal association related to CAM use and pregnancy. Secondly, a survey data is based on self-report and therefore prone to recall bias.

## 5. Conclusions

Evidence from this study suggests that a significant proportion of pregnant women were utilizing CAM including herbal products as part of their maternity care. The most obvious finding to emerge from this study is that a considerable number of women failed to disclose the use of CAM modalities for HCPs. These findings provide a reference on the use of CAM in pregnancy for policy-makers, health professionals, and parents. Further studies are warranted to explore the effectiveness and safety of CAM therapies like herbal medicinal products and potential effects of long-term use as well as possible interactions of herbal medicinal products with concomitantly used conventional medicines.

## Figures and Tables

**Table 1:** Sociodemographic characteristics of pregnant women and their association with CAM use in univariate and multiple logistic regression models (*N* = 282).

Characteristics	Overall *N* (%)	CAM use *N* (%)		*P* value *^a^*	COR (95% CI)^∗^	*P* value *^a^*	AOR (95% CI)^∗^
		Yes	No				
*Age (year)*							
<20	17 (6)	15 (6.0)	2 (6.7)		**1 (ref)**		
20-30	176 (62.4)	156 (61.9)	20 (66.7)	0.96	0.96 (0.20-4.52)	—	—
>30	89 (31.6)	81 (32.1)	8 (26.7)	0.71	0.74 (0.14-3.83)	—	—
*Religion*							
Orthodox	222 (78.7)	200 (79.4)	22 (73.3)	0.123	0.11 (0.007-1.82)	—	—
Muslim	55 (19.5)	48 (19.0)	7 (23.3)	0.99	—	—	—
Protestant	3 (1.1)	3 (1.2)	0 (0.0)	0.19	0.15 (0.008-2.60)	—	—
Catholic	2 (0.7)	1 (0.4)	1 (3.3)		**1 (ref)**		
*Marital status*							
Married	256 (90.8)	232 (92.1)	24 (80.0)		**1 (ref)**		**1 (ref)**
Single	20 (7.1)	15 (6.0)	5 (16.7)	**0.036**	3.22 (1.07-9.64)	0.18	2.39 (0.663-8.65)
Divorced	4 (1.4)	3 (1.2)	1 (3.3)	0.31	3.22 (0.32-32.19)	0.32	3.50 (0.28-43.20)
Widowed	2(0.7)	2 (0.8)	0 (0.0)	0.99	—	0.99	—
*Educational*							
Unable to write and read	78 (27.7)	75 (29.8)	3 (10.0)	**0.04**	0.20 (0.046-0.93)	0.14	0.29 (0.05-1.53)
Able to write and read	44 (15.6)	38 (15.1)	6 (20.0)	0.76	0.82 (0.22-2.97)	0.95	1.03 (0.26-4.10)
Primary school	53 (18.8)	50 (19.8)	3 (10.0)	0.13	0.31 (0.069-1.40)	0.30	0.44 (0.09-2.11)
Secondary school	76 (27)	63 (25.0)	13 (43.3)	0.90	1.07 (0.34-3.31)	0.96	1.02 (0.31-3.31)
College/university	31 (11)	26 (10.3)	5 (16.7)		**1 (ref)**		**1 (ref)**
*Occupation*							
Unemployed/house wife	162 (57.4)	147 (58.3)	15 (50.0)	0.37	0.54 (0.14-2.08)	—	—
Part time	43 (15.2)	40 (15.9)	3 (10.0)	0.29	0.40 (0.07-2.19)	—	—
Full time	58 (20.6)	49 (19.4)	9 (30.0)	0.97	0.98 (0.23-4.06)	—	—
Student	19 (6.7)	16 (6.3)	3 (10.0)		**1 (ref)**		
*Monthly income (Ethiopian birr)*							
No income	20 (7.1)	20 (7.9)	0 (0.0)	0.99	—	—	—
<500	86 (30.5)	82 (32.5)	4 (13.3)	0.19	0.25 (008-0.79)	—	—
500-999	83 (29.4)	72 (28.6)	11 (36.7)	0.59	0.79 (0.34-1.84)	—	—
>1000	93 (33)	78 (31.0)	15 (50.0)		**1 (ref)**		
*Previous number of children*							
No	108 (38.3)	92 (36.5)	16 (53.3)	**0.05**	3.30 (0.92-11.84)	0.42	1.82 (0.41-8.05)
1	60 (21.3)	52 (20.6)	8 (26.7)	0.12	2.92 (0.73-11.60)	0.31	2.16 (0.48-9.71)
2	54 (19.1)	51 (20.2)	3 (10.0)	0.89	1.11 (0.21-5.78)	0.73	0.73 (0.12-4.31)
≥3	60 (21.3)	57 (22.6)	3 (10.0)		**1 (ref)**		**1 (ref)**
*Pregnancy status*							
1^st^ trimester	55 (19.5)	47 (18.7)	8 (26.7)		**1 (ref)**		
2^nd^ trimester	124 (44)	109 (43.3)	15 (50.0)	0.65	0.80 (0.32-2.03)	—	—
3^rd^ trimester	103 (26.5)	96 (38.1)	7 (23.3)	0.12	0.42 (0.14-1.25)	—	—
*Antenatal care*							
No	47 (16.7)	39 (15.5)	8 (26.7)		**1 (ref)**		**1 (ref)**
Midwife	209 (74.1)	191 (75.8)	18 (60.0)	**0.05**	0.45 (0.18-1.13)	0.62	0.77 (0.27-2.18)
Nurse	10 (3.5)	8 (3.2)	2 (6.7)	0.82	1.21 (0.21-6.84)	0.39	2.30 (0.34-15.59)
Physician	16 (5.7)	14 (5.6)	2 (6.7)	0.67	0.69 (0.13-3.68)	0.93	1.08 (0.18-6.50)
*Residency*							
Urban	241 (85.5)	214 (84.9)	27 (90.0)	0.45	0.62 (0.18-2.16)	—	—
Rural	41 (14.5)	38 (15.1)	3 (10.0)		**1 (ref)**		

CAM: complementary and alternative medicine; ^a^*P* value refers to the significance of the differences between CAM users and non-CAM users using the chi-square test; COR: crude odds ratio; AOR: adjusted odds ratio; CI: confidence interval; ^∗^odds ratio and their 95% CI were derived using a binary logistic regression model with CAM use as the dependent variable.

**Table 2:** The observed responses for the use of CAM in the study participants (*N* = 252).

Queries	Overall *n* (%)
*Responses to quality of life assessment* ^a^	
Is CAM therapy improving your QOL?	86 (30.5)
My overall health is better	162 (57.4)
I am not depressed all the time	126 (44.7)
I have less fatigue	176 (62.4)
I have more energy	78 (27.7)
I can sleep well	185 (65.6)
I have better memory	162 (57.4)
I do not have frequent nausea/vomiting or other health problem	83 (29.4)
I am not sick all the time	75 (26.6)
*Disclosure of CAM use to HCPs*	
Yes	95 (33.7)
No	157 (55.7)
*Reasons for not reporting to HCPs* ^a^	
Does not need the HCP's approval	7 (2.5)
The HCPs would be unsupportive and would discourage	52 (18.4)
Insufficient information of CAM	19 (6.7)
The HCPs do not ask about CAM	57 (20.2)
*Source of information on CAM use* ^a^	
Family/friend/relative	140 (49.6)
Media (internet/television/radio/book)	16 (5.7)
Health practitioner	40 (14.2)
Pregnant women who used herbal medicines	12 (4.3)
CAM practitioners	18 (6.4)
*Awareness of drug-herb interaction*	
Yes	94 (33.3)
No/I do not know	158 (56)
*Satisfaction level with CAM use*	
Satisfied	59 (20.9)
Average	125 (44.3)
Dissatisfied	68 (24.1)

CAM: complementary and alternative medicine; QOL: quality of life; HCPs: healthcare providers; ^a^the values do not sum up to 100% since multiple-choice answers could have been selected.

**Table 3:** Reasons for using CAM^†^ in pregnancy (*N* = 252).

Reasons for using CAM	Responses *N* (%)		
	Strongly agree	Agree	Disagree
Beneficial effect on quality of life and health^a^	43 (15.2)	99 (35.1)	110 (39.0)
Its more natural	49 (17.4)	151 (53.5)	52 (18.4)
CAM is accessible and available	79 (28.0)	121 (42.9)	52 (18.4)
Improves nutritional status	12 (4.3)	64 (22.7)	176 (62.4)
Less risk for fetus than convectional drugs	20 (7.1)	63 (22.3)	169 (59.9)
Lack of trust on conventional treatments	9 (3.2)	52 (18.4)	191 (67.7)
Dissatisfaction with conventional therapy	6 (2.1)	62 (22.0)	184 (65.2)
Family/culture/religious beliefs	62 (22.0)	127 (45.0)	63 (22.3)
Previous experience of CAM use	21 (7.4)	120 (42.6)	111 (39.4)
Control over situation^b^	13 (4.6)	78 (27.7)	161 (57.1)

CAM: complementary and alternative medicine; ^†^the values do not sum up to 100% since data from CAM nonusers have not been selected; ^a^beneficial effect on quality of life and physical health of their fetus; ^b^CAM therapies gave a sense of control or autonomy over the situation.

## Data Availability

The materials and data supporting the findings of this study are available from the corresponding author upon reasonable request.
